# Values in Psychometrics

**DOI:** 10.1177/17456916211014183

**Published:** 2021-11-30

**Authors:** Lisa D. Wijsen, Denny Borsboom, Anna Alexandrova

**Affiliations:** 1 Department of Psychology, University of Amsterdam; 2 Department of History and Philosophy of Science, King’s College, University of Cambridge

**Keywords:** values, psychometrics, value-free ideal, objectivity, quantification, validity, fair test, utility

## Abstract

When it originated in the late 19th century, psychometrics was a field with both a scientific and a social mission: Psychometrics provided new methods for research into individual differences and at the same time considered these methods a means of creating a new social order. In contrast, contemporary psychometrics—because of its highly technical nature and its limited involvement in substantive psychological research—has created the impression of being a value-free discipline. In this article, we develop a contrasting characterization of contemporary psychometrics as a value-laden discipline. We expose four such values: that individual differences are quantitative (rather than qualitative), that measurement should be objective in a specific sense, that test items should be fair, and that the utility of a model is more important than its truth. Our goal is not to criticize psychometrics for supporting these values but rather to bring them into the open and to show that they are not inevitable and are in need of systematic evaluation.

Psychometrics—the discipline concerned with the measurement and prediction of psychological traits, aptitudes, and behavior—plays a central role in scientific psychology, educational measurement, and the structure of our current society (Borsboom & Wijsen, 2017; Lemann, 1999). Despite the evident social relevance of psychometrics, however, it is typically regarded as a largely technical discipline organized around the analysis of psychometric test data. This portrayal of psychometrics, as a conventional academic discipline whose primary goals appear to be purely scientific, makes it harder to detect any deeper political or social allegiances. This is remarkable because psychometrics has traditionally been deeply invested, through the work of its founding figures, in social and political projects, such as the eugenics movement, the introduction of military testing during the world wars, and the rise of a national education system (Cerezo, 1991; Evans & Waites, 1981; Jones & Thissen, 2007; Sokal, 1987). As a result, its methods and tools have been explicitly geared toward furthering the goals of these movements. So has psychometrics shaken off its ideological feathers? In keeping with recent work in the history and philosophy of science, which has uncovered value judgments across many areas of applied and theoretical science, we argue that it has not. Despite its technical appearance, social and ethical allegiances exist even in the most technical areas of psychometrics, right down to the structure of some of its central mathematical formulae. These allegiances mean that psychometrics is a socially and politically value-laden scientific discipline, and its statistical methods and assumptions partly reflect value systems and should be evaluated as such.

As with all concepts, the meaning of *psychometrics* is fluid and fuzzy and has no clear boundaries. When we speak of psychometrics in this article, we do not intend to cover psychometrics in all its shapes and forms. To focus the discussion, therefore, we adopt a specific conceptualization of psychometrics: Psychometrics as discussed here is the discipline concerned with the construction of abstract statistical models that can be used in psychological or educational research but have no particular interpretation in terms of specific psychological attributes (in contrast to, e.g., mathematical psychology, which constructs models for specific psychological systems). Thus, as the terminology is intended in this article, psychometricians tend to work on the technical aspects of psychometric models, as used in item-response theory (IRT) and structural equation modeling (SEM). The Psychometric Society is the institution that best represents this field, and *Psychometrika*, its flagship journal, is the primary vehicle for publishing research on the technical or statistical features of psychometric models. Models in these areas of research typically depict the relationship between a latent variable, which may represent any psychological construct, and the items that this variable purportedly measures. To draw a sharp contrast, psychometrics, as we understand the term here, mostly focuses on developing the technical aspects of these models (e.g., model extensions, methods for parameter estimation, methods for model fit assessment). This work is not to be confused with the application of these models to a construct of interest (e.g., cognitive abilities, personality dimensions, or psychological disorders) and its relation to other psychological or social attributes. For the sake of clarity, we call this subfield “psychometrics” in the remainder of this article, all the while recognizing that not all people who identify themselves as psychometricians may share a focus on this strictly technical approach.^1^

What does it mean to say that psychometrics is or is not value-laden? Value judgments in science are typically understood to be those explicit claims or implicit commitments that enable inference and theorizing where empirical or logical constraints do not uniquely compel any answer and where a wider range of considerations get deployed to advance research programs (Longino, 1996). Some value judgments are claims about what inference or theory is most predictive, most explanatory, or empirically adequate; these are known as *epistemic values* because they are values that relate primarily to the pursuit of knowledge. Value judgments about what is considered “good,” “right,” “just,” or “beautiful” are known as *nonepistemic values* (Douglas, 2000, 2009; Rooney, 1992). According to proponents of the value-free ideal, nonepistemic values do not have a place in science because they are not constitutive of knowledge or truth. However, philosophy of science recognizes a variety of ways in which epistemic and nonepistemic value judgments enter both pure and applied scientific research at all stages, from research planning to hypothesis testing (Douglas, 2009; Elliott, 2017). In the current article, we argue that this characterization also holds for psychometrics. Combining these terminological conventions, we speak of psychometrics as value-laden in the sense that the practice of psychometrics, whether theoretical or applied, routinely involves the use of value judgments. More specifically, we use the term “value-laden” to pick out two features: (a) Value judgments have a nonepistemic as well as epistemic character, and (b) value judgments enter into the construction and appraisal of psychometric models and hypotheses, not just prior decisions about what to research or how to apply existing knowledge. Thus, when we speak of psychometrics as having a “value-free” image, we mean that it denies (or fails to recognize) these two features.

Values in psychological science have been addressed on a number of occasions. For example, some studies have investigated the need for a diversity of political preferences in academic psychology (Duarte et al., 2015; Redding, 2001); other studies have investigated how psychology can become a more socially engaged, or socially conscious, discipline (Gergen, 1973; Nafstad & Blakar, 2012). Several studies have also addressed the role of values in the process of educational measurement and standardized testing. These studies were normative endeavors strongly laden with judgments about what is worthy, proper, or suitable, especially in high-stake situations (e.g., college admissions) or when those qualities themselves are considered especially desirable (Stein, 2014). Moral values are also strongly visible in what we consider “prepsychometrics”: the process of deciding which abilities and aptitudes are important and in need of measurement (a process that lies more in the hands of policymakers and applied psychologists than in the hands of psychometricians themselves). Values in the realm of testing, such as the ideas of social justice or equal opportunity, have been discussed by several authors (Gordon & Terrell, 1981; Messick, 1975, 1989). However, the role of value judgments in contemporary psychometrics, and specifically how they are embedded in much of the technical aspects of psychometric research, has not been explored in the literature so far and is our focus in this article.

Our strategy is to distinguish and illustrate four senses of value-ladenness in contemporary psychometrics: the conceptualization of individual differences as quantitative (rather than qualitative), the aim for objectivity in measurement, the aim for fairness in measurement, and the preference for utility above truth. All four might look like scientific commitments rather than value judgments. We argue that, when commitments such as objectivity (i.e., the removal of personal judgment) are endorsed in psychometrics, this is not evidence of value freedom. Instead, these commitments in part embody the endorsement of a specific social value, such as seeking to remove personal judgment—as much as possible—from the testing process and to substitute it with a combination of good assessment procedures and proper data analysis. In doing so, today’s methods of modeling are most naturally aligned with the ideal of a meritocratic society in which people’s role in society can be assigned on the basis of measurable characteristics pertaining to their abilities. We should thus resist the temptation to view these value judgments as somehow inevitable, inherent, and fundamental, such that psychometrics would not be psychometrics without them. Each of these value judgments is contingent, rather than necessary, and each of them could very well have been different. To illustrate this point, we trace the historical reasons for adopting each of the four and show that there were and still exist precedents for alternative value judgments.

In articulating the ways in which even the most technical work in psychometrics involves value judgments, our goal is not to criticize the discipline or to expose it as somehow biased and failing to live up to the ideals of science. Any inquiry needs to make the sort of foundational bets we attribute to psychometrics, for without them the scientific work cannot get started. However, exactly what commitments a discipline adopts are neither inevitable nor innocent. Making them explicit will hopefully stimulate more discussion on the implementation of moral values in psychometric research and thereby contribute to a more reflective psychometric practice in psychological science and society.

## “Value-Free” State-of-the-Art Psychometrics

Before we show how social values permeate psychometric research, we first discuss the methodology we adopt and explain why contemporary psychometrics cultivates a value-free image. A full investigation of values in this field, even when defined in our narrow sense, would require a wide array of evidence (e.g., interviews, survey data, citation analysis). As a starting point for such an investigation, our contribution adopts a methodology common in philosophy of science, in which values of a field are inferred from the commitments implicit in its exemplary projects and theories.^2^ This does not mean that individual psychometricians hold these attitudes at a personal level but only that these commitments underlie their theoretical work and without these commitments this work does not make sense. Likewise, when we discuss the value-free image of psychometrics, we mean the shared impression that psychometrics is free of those commitments. There are of course limits to this methodology because it does not pick up on the attitudes of individual psychometricians that cannot be deduced from their publications. We also cannot speak for the entire scope of psychometrics. Nevertheless, because we show that values are already detectable at this very theoretical and technical level of psychometric research, we hypothesize that they will also be more easily detectable at more applied levels.

Exposing the values in psychometrics means putting pressure on its value-free self-conception. There are three observable manifestations of this self-conception. First, psychometric research tends to focus on the technical and statistical features of psychometric models rather than substantive interpretations of these models. The modeling traditions of IRT and SEM are two of its main focus areas, and examples of important projects in contemporary psychometrics include computerized adaptive testing, a form of computer-based testing that adapts the items to the examinee’s ability level, often on the basis of IRT principles; Bayesian estimation of psychometric models; methods for model evaluation and comparison; and response-time analysis. *Psychometrika*, psychometrics’ flagship journal, is a mainly theoretical and technical journal that emphasizes the statistical aspects of (often highly specialized) methods and models, and articles in *Psychometrika* are known to be fiendishly technical and difficult to understand for researchers who lack advanced statistical or psychometric education. Although there have been efforts to open up *Psychometrika* to a larger, more applied audience (e.g., *Psychometrika* now has an application reviews and case studies section), its coverage has remained largely devoid of psychological content. Articles in *Psychometrika* focus on the technical and statistical aspects of models and research methods rather than specific substantive interpretations. Correspondingly, latent variables, once considered innate “measurable” entities (Spearman, 1904), are now often considered purely statistically (e.g., as random effects or as summaries of the data; Jonas & Markon, 2016), convenient for the process of modeling and estimation but not as a reference to an existing psychological attribute. *Psychometrika* is a journal that contains articles written by specialist authors for specialist readers; although individual authors and editors may labor to make its content accessible to a wider audience, such accessibility is clearly not a core goal of the journal. In contrast, *Psychological Methods*, a journal that publishes a wide variety of article types on quantitative methods in psychology (among which is psychometric methods but not exclusively so), explicitly encourages authors to make their work understandable to applied researchers.

Because of *Psychometrika*’s focus on technical issues, psychologists and other applied researchers may be inclined to leave the highly technical psychometric literature for what it is; in turn, psychometricians may not always be sensitive to the problems of applied research (Sijtsma, 2009; Young, 1996). This reciprocal lack of involvement with the other party contributes to the status of psychometrics as a field without a very strong connection to substantive psychological research. And being such an abstract and technical field has made the detection of an explicit political agenda (if there is any) in this research difficult. Contemporary psychometric research thus invites the impression of being a field with no apparent social mission and with only technical tools to offer. This impression motivates the idea that contemporary psychometrics is value-free.

A second manifestation of psychometrics’ apparent value freedom is shown by the departure of *validity*—the core term that connects psychometric models to substantive applications (Borsboom & Markus, 2013; Cronbach & Meehl, 1955; Kane, 2006; Messick, 1989)—from the psychometric jargon used in journals such as *Psychometrika*. Validity became a frequently used term in psychometric literature in the 1910s and 1920s (Newton & Shaw, 2014) and was conceptualized by psychometrician Truman Kelley (1927) as “whether a test really measures what it purports to measure” (p. 14). Witnessing the rise of intelligence tests, Kelley found it important to be careful about drawing bold conclusions about a person’s attributes and argued that it was first and foremost important to know whether a test indeed measures what it aims to measure before one draws these conclusions. The definition of validity has gone through a number of changes, and several contemporary definitions emphasize that validity is not only a matter of whether a test actually measures the construct of interest (i.e., test validity; Borsboom et al., 2004) but also a matter of whether sufficient evidence supports specific interpretations and uses of a test (Kane, 2001).

Although psychometricians still care about validity in a general sense and would agree that it is of vital importance that measures are thoroughly checked for validity, validity is no longer a common research topic in contemporary psychometrics. For example, between 2000 and 2019, only one original article and one book review in *Psychometrika* were devoted to validity (compared with hundreds in other psychology journals), whereas between 1940 and 1959, more than 15 articles in *Psychometrika* dealt with the topic of validity. So although validity is possibly one of the best known and most exported concepts in psychometrics and still of value to psychometricians themselves, the discussion of what validity entails and how to establish it has largely moved away from psychometrics proper. Instead, this debate takes place in educational and psychological research (e.g., Kane, 2001; Moss, 1995) and philosophy of science (e.g., Alexandrova & Haybron, 2016; Markus & Borsboom, 2013; Stone, 2019). The departure of validity from psychometric discourse could be interpreted as a sign of psychometrics’ increasingly instrumentalist approach.

A third aspect of contemporary psychometrics’ apparent value freedom resides in the fact that it does not endorse particular uses of testing for social purposes and does not recommend any particular educational or workplace policies. The debate on the role of tests and assessments in society is part of public discourse both in the United States and the Netherlands (two of psychometrics’ strongholds) but is mostly led by policymakers and education reformers (not by psychometricians). Psychometricians certainly work for institutions that promote standardized testing, such as the Educational Testing Service (ETS) in the United States or the Centraal Instituut voor Toets Ontwikkeling (CITO) in the Netherlands, but do not often engage in the debate on whether standardized testing is indeed a desirable development (a debate that is now again in full swing with the University of California, Los Angeles—among other schools—having rejecting the SATs as part of the admissions procedure). The lack of participation of psychometricians is to be expected given that historians of the field attribute to it an ideal called “technocratic rationality” (Evans & Waites, 1981). According to Evans and Waites (1981), this ideal commands that any problems be solved within the framework of the technology itself. Seen as technocratic rationality, psychometric research focuses on mostly technical and statistical problems that need to be solved and does not concern itself with long-term goals in terms of desirable social, economic, and political development. A more recent illustration of this idea is an argument by psychometrician Klaas Sijtsma (2006) that psychometrics cannot “replace substantive theorizing about intelligence or personality for designing good measurement instruments; it can only give support” (p. 454). The focus of psychometrics as a discipline is thus in solving the technical parts and with that supporting the psychologists or applied researchers rather than engaging in substantive theory or the social and political interests that follow from their work.

The apparent value freedom of contemporary psychometrics thus follows from psychometrics’ highly technical content, its somewhat isolated existence from applied research, and its tendency to operate within the constraints of technocratic rationality. Crucially for our argument the field has not always entertained or cultivated a value-free image. Early psychometricians and founders of the field, such as Francis Galton, Lewis Terman, James McKeen Cattell, and Charles Spearman, often incorporated explicit commitments about how psychometrics could and should be used for certain social or political purposes. For example, following a eugenic ideology that many early psychologists upheld, psychometric measurement was considered a tool for setting up the “ideal” society, in which intelligence measurement would play an incremental role in setting up a meritocratic hierarchy, eliminating crime, and deciding who was encouraged to procreate and who was discouraged from doing so. The shift that we see here—from a psychometrics that is committed to larger political projects to a psychometrics that is a largely technical discipline without such explicit commitments—shows that psychometrics has not always abstained from making value commitments and that the use and interpretation of particular values does not follow naturally from the discipline itself. In this article, we argue, however, that the value-free image of contemporary psychometrics is incorrect and that values are in fact woven into both early and contemporary psychometric research. We show how different values permeate and inform the practice of psychometric research and illustrate how the particular use of values in psychometrics is never an inevitable consequence but a choice of the researchers in a particular discipline.

## Values in Psychometrics

To show that the value-free conception of psychometrics is inaccurate, we discuss four values that permeate psychometric decision-making in different ways: the conceptualization of individual differences as having a quantitative (not qualitative) structure, the aim for objective measurement, the formalization of fairness of items, and the preference of utility above truth. Although we treat these values separately, there are areas of overlap that we highlight as we go through the analysis. Our strategy is first to show that a widely accepted method or a common assumption of psychometrics exhibits one or more of the value judgments above and second, using examples from the history of psychometrics, to emphasize that this particular value judgment is not inevitable and that there exist precedents for alternative values.

### Individual differences are quantitative, not qualitative

Psychometrics is traditionally committed to investigating individual differences in a quantitative fashion. Individuals are typically considered to be ordered by their values on a measured attribute, and the differences between them are quantitative—that is, if two individuals differ in intelligence or a scholastic aptitude such as reading ability, one of them is assumed to have more of it than the other rather than having a different *kind* of ability (Michell, 2000). Psychometric models, and especially Rasch models,^3^ clearly reflect this assumption (Bond & Fox, 2015), and this commitment has not significantly changed over time. In fact, for the most part, psychometrics is still dedicated to developing models for quantitative measurement. We argue that the choice for conceptualizing individual differences as quantitative rather than qualitative involves two values: the value that only quantitative knowledge counts as properly scientific and a moral commitment to a specific form of equality (i.e., that differences between human beings are only of degree and not of kind). Let us illustrate each in turn.

The “quantitative imperative” holds that proper science is always quantitative and is often epitomized in this quote from Lord Kelvin:

When you can measure what you are speaking about, and express it in numbers, you know something about it; but when you cannot measure it, when you cannot express it in numbers, your knowledge is of a meagre and unsatisfactory kind. (Thomson, 1889, p. 91)

The quantitative imperative has been argued to have influenced early psychology (Michell, 2003). This is visible in the work of early psychometricians. For example, Francis Galton believed that the only means to a scientific approach to the study of the mind is the measurement of quantitative differences (Galton, 1879). Quantitative research had proven successful in the natural sciences, and psychometricians were confident that it would also become the method for investigating laws of the human mind. McKeen Cattell (1890) expressed this ardent belief:

Psychology cannot attain the certainty and exactness of the physical sciences unless it rests on a foundation of experiments and measurement. A step in this direction could be made by applying a series of mental tests and measurements to a large number of individuals. (p. 373)

The view that a quantitative approach makes research more rigorous and reliable, and thus more scientific, has since been a guiding principle of much psychometric research.^4^ The journal *Psychometrika* is still “devoted to the development of psychology as a quantitative rational science” (Psychometrika, n.d., para. 1), and most articles in *Psychometrika* concern quantitative and statistical methods. By conceptualizing abilities and aptitudes as quantitative attributes and by developing quantitative methods to analyze and measure these abilities, both early and contemporary psychometricians have aimed at drawing what they saw as uniquely reliable conclusions about individual differences.

It may be tempting to view the quantitative assumption as somehow inevitable and inherent to psychometrics, such that psychometrics would not be itself without it. We resist this temptation. There have been a number of more qualitatively oriented approaches in psychometrics worth mentioning (although these still rely on statistical methods and are not qualitative in the sense of gathering nonnumerical data). One example is Thurstone’s theory of primary mental abilities, which assumes that intelligence does not consist of one factor but multiple independent factors (Thurstone, 1938). Although people differ on these factors in terms of degree, there is also qualitative differentiation: Every person has an individual “profile” of levels on the different factors; that is, there is a pattern of scores rather than a single score. A second example is the Aptitude × Treatment interaction that investigates how different methods of instruction interact with student ability and achievement (Cronbach & Webb, 1975); in certain cases, these differences can be qualitative in the sense that the functional form of intervention effects depends on the aptitude (e.g., when a teaching method benefits able students but has adverse effects on less able students). There has also been the occasional psychometric model for qualitative differences that assumes a discrete rather than continuous latent variable (e.g., the latent-class model). A class in such a model accounts for a specific pattern of item responses. These models, however, are not mainstream in psychometrics (only two articles in the 2018 and 2019 issues of *Psychometrika* addressed latent class models, and the assumption that individual differences are quantitative still form the vast majority of *Psychometrika* articles).^5^ Perhaps unsurprisingly, the latent class model originated in the work of sociologists Lazarsfeld and Henry (1968), not in psychometrics, and Cronbach and Webb (1975) was published in the more theory-oriented *Journal of Educational Psychology*, not in the more technical *Psychometrika*. All in all, it is safe to state that the assumption of quantity has won out in psychometrics, although there have been dissenters.

So far so uncontroversial. Less appreciated is that, in addition to being a commitment about proper scientific method, the commitment to quantity also has a moral dimension—in particular, the commitment to quantification can be seen as a commitment to a form of equality. This moral component becomes clear when we consider there were times when differences between people were characterized qualitatively to indicate inferiority of certain groups. An 18th-century U.S. slave owner would not have agreed with the idea that the difference between him and his slave is a difference in degree. The slave after all was considered a kind of animal, an entirely different species that was inferior to the White man (especially with regard to cognitive abilities).^6^ Likewise, the Nazis typically held qualitative differences in high regard: They considered Jewish and Roma people to be subhuman (Steizinger, 2018). To say that the difference between people is a matter of degree, rather than of kind, is to negate these absolute differences. In this sense, it codes the idea that, in essence, we are all equal. The sense of equality here is thus the fundamental equality in the kind of creatures we are and hence the endowments we all have, although the levels of these endowments can differ person by person. This may sound ironic because a core goal of psychometric applications is to differentiate people in rankings (one of its core parameters is literally a discrimination parameter that indicates how well particular items perform in this respect), which typically results in *unequal* outcomes for those involved. Moreover, it is very hard to attribute a commitment to equality to the early practitioners of psychometrics who were in search of a superior race and a superior gene pool. This irony notwithstanding, today’s psychometricians adhere to a particular type of equality in which (cognitive) differences between people are a matter of a degree, while at the same time rejecting the embarrassing allegiances of their predecessors.

In addition to the moral and the epistemic justifications for quantification, there is also a deeply practical one: Numerical rankings enable psychometricians to give pithy summaries of enormous amounts of data and information about individuals. As we illustrate later, the commitment to being “practical” is another crucial commitment of contemporary psychometrics. Comparing people’s abilities on a quantitative scale is highly practical and often more effective than if we had conceptualized differences between people qualitatively. Decisions based on qualitative data are typically hard to trace, not very transparent, and thus do not easily lend themselves to psychometric analysis. This aligns with Porter’s well-known argument that quantification “minimizes the need for intimate knowledge and personal trust” (Porter, 1995, p. ix) and aids communication about the objects of this quantification beyond the community itself. We can directly compare students on the measured trait, observe who has a higher level of intelligence or other cognitive skills, and then decide who is accepted into college and who is not.

The commitment to quantity in psychometrics is not one justified to everyone’s satisfaction. It has been argued that there is no strong evidence that attributes are in fact quantitative in nature, although psychometricians treat psychological attributes as such (Michell, 1999, 2000, 2008). Michell argued that the stance of the psychometrician here is even pathological: Psychometricians ignore the alternative hypothesis that an attribute is not quantitative, which is a sign of bad science. We do not wish to argue here that psychometrics is indeed a pathological science (for a counterargument, see Borsboom & Mellenbergh, 2004), but what this angle does show is that it is not a priori the case that psychological attributes should be treated in a quantitative fashion.

Michell (2019) elaborated on the interesting case of philosopher R. G. Collingwood, who was a strong opponent of the dominant quantitative stance in psychophysics and psychometrics. Collingwood argued that a psychological attribute such as heat sensations can be seen as a combination of degree and kind as heat experience varies “from a faint warmth through a decided warmth it passes to a definite heat, first pleasant, then dully painful; the heat at one degree soothes me, at another excites me, at another torments me” (Collingwood, 1933, p. 72). Whereas the heat increases by degree (Collingwood did not deny that many attributes in physics differ quantitatively), our heat sensations differ in kind (our experience is not only that the temperature rises but also that it goes from a pleasant sensation to an uncomfortable and then highly painful sensation). Our point here is not to argue that Collingwood was right and that psychometricians were wrong. From a psychometrician’s point of view, there are certainly several possible reasons why a qualitative conceptualization would be morally, scientifically, and practically undesirable. For example, how would one build a fair and reliable measurement procedure if one can no longer compare people on a single scale? However, Collingwood’s example here shows that, regardless of these objections, the quantification of psychological attributes does not follow naturally from some objective reality psychometricians aim to describe but also that there is an alternative history of psychology and psychometrics imaginable with a weaker (or perhaps even lack of) emphasis on individual differences as quantitative differences.

In sum, the quantification of individual differences has both a moral and an epistemic component: The moral component is the idea of equality of standards, and the epistemic component is the centrality of quantification to rigorous science. The strong adherence to quantification in psychometrics also enables the psychometrician to claim a very specific ideal of objectivity, to which we turn now.

### Objectivity

Objectivity is perhaps the quintessential epistemic value in all sciences, whether natural or social. Work in the history and philosophy of science shows that objectivity has several meanings, and which of these meanings is prized is highly contingent on the history and the context (Daston & Galison, 2007; Douglas, 2004). Objectivity can mean being true to nature from God’s point of view, restricting the personal preferences or desires of the inquirer, or simply following transparent and mechanical procedures (e.g., the open-science movement; Nosek et al., 2015). There is no one true definition, and exactly which of the many definitions a discipline settles on can reveal a great deal about its identity. Psychometrics is no exception, and the definition of objectivity it adopts is motivated by considerations that are very specific to psychometrics: This field places a huge premium on avoiding the risk that a personal judgment taints the measurement and testing process (i.e., how performance is mapped to a dimension).^7^ The personal judgments by individuals can be informed by factors that have little to do with an examinee’s ability, such as the examinee’s gender, religion, or social class, or simply by a general dislike for the examinee. Psychometricians reject all evaluations in which teachers exclusively rely on their own judgment rather than on objective sources such as test scores. To ensure objectivity in this sense psychometricians develop assessment procedures that control for possible biases.

The importance of objectivity in the history of psychometrics can be seen in the work of Spearman, whose mission was to contribute to “psychology of a more exact character,” in which abilities can be “definitely measured and permanently recorded” rather than being estimated by “hearsay, causal experience, and remote reminiscence” (Spearman, 1914, p. 25). Spearman was a member of the Eugenic Society and considered general intelligence to be the basis of social institutions (Spearman, 1914, 1927). Spearman was the first to use the quantitative Galtonian and Pearsonian techniques, such as correlation and regression, for modeling intelligence. In his 1904 article, appropriately called “‘General Intelligence,’ Objectively Determined and Measured,” Spearman formalized the relationship between general intelligence (*g*) and the test items that are supposed to measure this attribute in his common-factor model. Spearman thus had a strong scientific curiosity to uncover the structure of the mind through the use of objective, statistical methods that did not rely on human judgment, and his common-factor model was a good match with the politics of eugenics: Spearman considered general intelligence to be a hereditary quality that resided in the brain (although he was unsure where exactly), and the common-factor model could contribute to measuring people’s intelligence in an objective fashion and to shaping a society that was ordered on the basis of this quality. In the words of Spearman himself:

Thus, it is to be hoped, we shall eventually reach our pedagogical conclusions, not by easy subjective theories, nor by the insignificant range of personal experiences, nor yet by some catchpenny exceptional cases, but rather by an adequately representative array of established facts. (p. 277)

The importance of objectivity for Spearman—making judgments about people’s level of skills on the basis of evidence rather than our faulty subjective reasoning—has since remained an important aspect of psychometric research and has received further scrutiny. In contemporary psychometrics, however, objectivity has received a more specific definition. According to Rasch modelers, for instance, the concept of objectivity is operationalized in terms of the interchangeability of sources of evidence because it holds that judgments about a person’s ability, and decisions based on these judgments, should be invariant under a particular change of perspective; namely, they should not depend on which items or raters are used to base the decision on. The requirement that conclusions should be invariant under exchanging observers, raters, interviewers, or items is made explicit in several formal definitions of psychometric models. For example, in the basic unidimensional latent-variable model (Mellenbergh, 1994), it does not matter (except for measurement error) which particular items are used to arrive at conclusions regarding the ordering of individuals (Grayson, 1988). Perhaps the best known instance of this property is Rasch’s concept of “specific objectivity” (Rasch, 1967, 1968, 1977): the requirement that the comparison of persons should not depend on which items are chosen and vice versa (see also Fischer, 1987). Likewise, Junker and Ellis (1997) conceptualized the idea of the “exchangeability” of psychometric items. Along similar lines, decisions should not depend on any particular person (i.e., a *rater*, in psychometric jargon; Cronbach et al., 1972). Objectivity in psychometrics thus means the removal, as far as possible, of any leeway and any freedom from the users and the administrators of a test, as well as the notion of specific objectivity or measurement invariance, in which the estimation of a person’s ability “must not depend on the items nor on the distribution and sampling of persons” (Kreiner, 2007, p. 281).

Because objectivity is often defined as the elimination of personal bias, it is tempting to see it solely as an epistemic value. However, it is also a nonepistemic value because objectivity in psychometrics takes away the power of judgment from individuals so that the power of judgment comes to rely on facts about a person’s ability rather than human judgment. Objectivity in psychometrics is thus a statement on where judgment *ought to* lie: with science. As a consequence, when a psychological construct becomes objectified it falls within the reach and under the technical control of psychometrics. It becomes an object at the psychometrician’s disposal. The objectivity-of-intelligence measurement opened the door to using it for a certain purpose, and in Spearmanian times, this meant creating a society in which people with high intelligence were encouraged to have offspring and discouraging others to do so. Although eugenic ideology no longer has a common place in our politics, a meritocratic ideology certainly thrives in the United States and most of the Western world, and we widely use cognitive and intelligence tests to facilitate this ideology. Psychometrics as a discipline relies for its prestige and standing on being part of these practices. In this sense, although objectivity denotes a departure from values, it enables psychometrics to participate in projects of moral and political valence. The story is structurally similar for fairness, to which we move to now.

### Fairness

Both values discussed above, the aim for objective and quantitative measurements, can be interpreted in terms of specific kinds of fairness. Not only should measurement take place in an objective fashion to avoid any value judgments from people involved, but also, ultimately, the most qualified people should be awarded the highest test scores and be granted whatever was at stake (e.g., the job position or enrollment at a university). However, in the second half of the 20th century, there was a growing awareness that people with the highest ability do not automatically gain the highest test score and that measurement instruments could in fact generate biases, even if the same items were used for all individuals.

In the first half of the 20th century, standardized testing was a means to support the eugenic beliefs of the time. The on-average lower test scores of minorities such as immigrants and African Americans were attributed to their inferiority to Whites and were considered “proof” of their lower potential and worth. Sources that could possibly explain these differences in test scores (such as lack of schooling or financial resources of specific groups or items that discriminate between groups) were largely ignored. In the second half of the 20th century, awareness of unfairness in standardized tests increased. One of the key moments was the *Larry P. v. Riles* (1979) case, in which a U.S. district court decided that standardized IQ tests were racially biased against African American children, who were, because of their lower scores, placed in inferior education settings that in turn only increased their isolation from a suitable high-quality learning environment. This and other rulings resulted in a complete ban against administering IQ tests to African American children for any special-education purpose (Frisby & Henry, 2016). *Larry P. v. Riles* and other lawsuits have contributed to the awareness among psychometricians that tests, and even items, can discriminate against specific groups (e.g., gender, cultural, or minority groups). Test or item bias became an important topic in psychometric research from the 1970s onward.

In psychometrics, an item is considered biased when respondents from different groups but with the same ability show different probabilities for answering an item correctly (Mellenbergh, 1989). The statistical concept developed to identify biased items, differential-item functioning (DIF), signals that the relevant item does not behave the same in different groups. When one or more items is affected by DIF and leads to adverse consequences for a specific group, DIF implies item bias. In the case of item bias, measurement is confounded by other (irrelevant) variables and thus cannot be considered completely objective in the psychometric sense (Kreiner, 2007). Psychometricians have developed various methods for identifying DIF (e.g., Holland & Wainer, 1993) and have written software packages so researchers could test for its presence (e.g., Shealy et al., 1991). Bias as conceptualized in psychometrics thus involves a restricted sense of fairness that pertains only to the fairness of specific items or tests and not to a wider social or political fairness (for a radically different conception of fairness, see, e.g., Broome, 1990).

In SEM, an important element in reaching fair decisions was formalized as *measurement invariance* (Meredith, 1993), which means that, across groups, the instrument relates to the latent variable in the same way. In other words, the same measurement model holds in different groups. Whereas DIF is usually item-specific (although there are instances in which groups of items rather than just one item are considered), measurement invariance denotes the complete absence of DIF in an entire test. Another example of fairness in psychometrics is test equating,^8^ which is another main tradition in psychometric research. Some tests are administered repeatedly (such as the SAT), and test scores of these tests should be comparable across versions. As with DIF and measurement invariance, psychometricians would argue that test equating should be evaluated by using statistical methods. Although testing agencies such as the ETS also use a variety of nonpsychometric methods when evaluating the fairness of a test, such as engaging external reviewers to help evaluate which items are unfair to marginalized groups, the methods mentioned above are central in evaluating the fairness of a test. Commentators on psychometrics have widely recognized these (and other) conceptualizations of fairness in testing practice (Dorans & Cook, 2016).

Although psychometricians have become more conscious of the possible unfairness in testing as illustrated by the many examples of tools to investigate fairness of tests, this has not led to a rejection of testing as such. Actually, the opposite is the case: Standardized testing, now including methods for calculating some statistic that detects possibly biased items, is still considered one of psychometrics’ main contributions to society (Wijsen, 2021). A society that is not aware of the problems of fairness in testing, or worse, a society that does not endorse testing at all, is not aware of the danger of its alternatives. After all, standardized testing excludes several sources of potential bias that would naturally enter our judgment, and it is likely that standardized testing has provided many people with opportunities that they otherwise would not have had.^9^

As explained above, psychometrics provides a definition of fairness that is based solely on the relationship between a test instrument and people’s abilities, which can certainly be seen as an emancipatory development: What matters is a person’s level of ability, not other irrelevant factors. This definition aligns well with our meritocratic society, in which we select people according to merit rather than to their financial situation, family reputation, religion, or other variables that should not influence selection criteria. However, the measurement-invariance definition as discussed above is not the only definition of fairness that is available in the literature and that has been put to use.

A second definition formulated by Cleary (1968) is that a test is unbiased if the regression of some criterion on the test scores is equal for different groups. For example, if the criterion of interest is success in the first year of college, and the predictors are the scores on an intelligence test, a fair test would find similar regression equations in different groups (e.g., men and women). According to this definition, it is not necessary that the same measurement model holds in different groups (which is necessary for the measurement-invariance definition of fairness). In fact, the measurement-invariance definition is inconsistent with the prediction-invariance definition: When the same measurement model holds in different groups (measurement invariance), the regression lines of the criterion regressed on test scores of these different groups will not generally be the same (Millsap, 1997, 2007). In accordance, as a rule, the quality of selection in terms of sensitivity and specificity cannot hold under measurement invariance if the latent-variable distributions differ across groups (Borsboom et al., 2008; Heesen & Romeijn, 2019). A third alternative for a definition of fairness would be that a test is unbiased when a selected group entails an equal (or representative) distribution of members from different groups. For example, when selecting first-year students for college entry, it could be decided beforehand that, when conditionalized on ability, there should be an equal number of men and women and a representative number of people from different ethnic backgrounds among the selected students. A test is then defined as unbiased when the criteria decided on beforehand hold.

There are conceivably several arguments for or against each of these definitions (Borsboom et al., 2008). Fairness defined in the sense of equal distribution of different groups is radically different from the way measurement-invariance fairness is defined: It accounts for the fact that some groups have fewer opportunities than other groups, and ability is not the only factor that determines whether someone is admitted. One cannot decide between these options on technical criteria alone, and whichever definition of fairness in psychometrics one upholds likely involves a moral evaluation of how measurement and selection should take place. This is undoubtedly a value judgment and one that is intimately connected to the practical role of psychometric research, to which we now turn.

### Utility above truth

The final value judgment on which modern psychometrics depends is the primacy of practical utility of its models over their theoretical virtues. This commitment also has a history showing that it is not inevitable or uncontroversial that psychometrics should prize utility above truth.

In addition to a genuine scientific interest in developing methods for the measurement of psychological attributes, many early psychologists and psychometricians also saw that psychometrics could potentially serve a social purpose. Psychometric instruments could in fact help arrange a new social order in society—an order with genetic merit. These beliefs were inspired by eugenic ideology, which was founded by one of psychometrics’ forebears, Francis Galton. According to eugenic ideology, a population could and should be improved by encouraging people with desirable traits to procreate and discouraging people with less desirable traits to do so.^10^ According to several educational psychologists, such as Henry Goddard and Lewis Terman, standardized testing could be considered a tool that identifies individuals with a higher eugenic worth (Stoskopf, 2002). The large-scale testing of immigrants at Ellis Island and military testing during World War I are both famous examples of how standardized testing was used to identify people with higher or lower eugenic worth. Shortly after the war, mental testing entered the educational system and was used to evaluate the abilities of millions of schoolchildren. According to Terman, who introduced the first intelligence test for American children in 1916 (the Stanford-Binet Intelligence Scales, a revision of the original French Binet-Simon scale) and devised several other measurement instruments over the years, mental testing served the American meritocratic democratic ideal. He argued that “the time has come when [intelligence testing’s] wider use for more general purposes should be encouraged” (Terman, 1916, p. xi). Intelligence testing should thus not remain only a scientific endeavor but one that could serve an important purpose in society as well.

The utility of psychometric tools was defined from this perspective as ensuring the welfare of American society, namely the education of people with high innate ability as picked out by mental tests (Minton, 1987). Moreover, tests were expected to “bring tens of thousands of these high-grade defectives under the surveillance and protection of society” (Terman, 1916, p. 7) so that crime and poverty could be eliminated. Psychologists and educational reformers considered mental testing the most effective method of reaching a meritocratic social order, both in the sphere of education by measuring intelligence of both the intellectually disabled and intellectually gifted and in the sphere of criminology to identify people who were weak-minded and more prone to engage in criminal activities. In the words of Minton (1987), “‘prediction and control,’ ‘human engineering’ and ‘social efficiency’ were the catchphrases for postwar American psychology” (p. 106). The eugenic ideology of Terman, Yerkes, and Goddard—shared by many psychologists and educational reformers at the time—inspired a significant reform in education in which standardized tests became the new normal.

From the very early onset of psychometrics, psychometrics was thus considered a practical tool that could be used for specific social or political purposes, and this utilitarian aspect of psychometrics (although no longer inspired by eugenic ideology) has not changed much. Contemporary psychometricians still greatly value research material that finds its place in one or more applications. In fact, the possibility of applying psychometric research is sometimes perceived as more valuable than uncovering fundamental knowledge and building theory that perhaps has no immediate connection to such applications (Borsboom, 2006; Sijtsma, 2006). Rather than trying to explain human behavior, much contemporary psychometric research answers very practical questions. Is this test fair with respect to test takers from different cultural backgrounds? What is the best method for estimating a person’s ability? How do we control for a set of confounds? How can we compare the abilities of schoolchildren from different countries? Exceptions aside (e.g., explanatory item-response theory; De Boeck & Wilson, 2004), most psychometric models usually do not aim at explanation. Psychometrics is thus relatively practical with regard to the question it answers and does not often seek a fundamental understanding of a mechanism. Knowledge for the sake of knowledge, or truth for the sake of truth, a value in many of the sciences, has gained little territory in contemporary psychometrics. It is important to note here that when we speak of utility above truth, it does not mean that psychometric research cannot indirectly contribute to psychological theory. Through psychometric models, psychologists can, for instance, formalize theories that explain human behavior, and a psychometrician might support the psychologist in that process, but for psychometric research itself, utility is more of a priority than truth.

To illustrate the utility value in psychometrics, we can draw a comparison between psychometrics and an engineering approach (Thissen, 2001; Wilson & Gochyyev, 2013)—a comparison that has also been suggested for the field of economics (Roth, 2002). Wilson and Gochyyev (2013) argued that, like engineering, psychometrics builds or constructs objects (the measures) and then develops models to analyze these measures, which psychometricians call “reverse engineering.” Psychometrics can thus be considered “a practical and engineering activity rather than as a basic science” (p. 3), in which basic science denotes seeking (fundamental) knowledge and understanding of mechanisms. Like engineering, one of the ultimate goals in psychometrics is that the research is used for building useful structures, such as new testing procedures, more advanced methods for data analysis in psychological science, or user-friendly software packages. And like engineering, these structures are considered more valuable than gaining fundamental knowledge of human psychology that has no direct link to practical output. The latter would be, in the case of engineering, the territory of the (theoretical) physicists, or in the case of psychometrics, the territory of the psychologists. This is not to say that there is no foundational research in psychometrics or that psychometrics does not indirectly contribute to foundational psychological knowledge. *Psychometrika* especially publishes many articles on foundational problems in psychological measurement, and the tools that psychometricians build can certainly be deployed to gain (psychological) knowledge. However, most foundational psychometric research is foundational in a statistical or mathematical sense rather than a psychological sense, and the way in which psychometrics contributes to psychological knowledge is often indirect: Psychometric models may be used for a variety of substantive issues but are not directly theoretically informed.

In addition to emphasizing building new structures that add on to the already elaborate testing technology, psychometricians have also become more focused on prediction and data analysis than on actual measurement. As mentioned previously, psychometricians themselves do not necessarily believe in the existence of psychological attributes or in the direct connection between reality and psychometric models (i.e., psychometricians do not often expect that psychometric models can or should be interpreted realistically). To illustrate this point, Sijtsma (2006) characterized latent variables as “summaries of the data, nothing more” (p. 452), and De Boeck (2008) considered them in a statistical fashion (i.e., as random effects). In other words, psychometricians often consider latent variables as properties of the data rather than being real entities. Although psychometricians might refrain from a realist interpretation, they consider psychometric models to be useful for the analysis of all sorts of behavioral data, which are increasing in number and volume. So rather than being restricted to measurement as it was originally intended, many psychometric models can be used for a wide array of data-analytic purposes. Because data analysis and prediction often have a practical motivation (e.g., data reduction or data visualization), the emphasis on data analysis and prediction in psychometrics aligns with the value of utility above truth.

The value of utility over truth is one of the factors that separate psychometrics from mathematical psychology, a different quantitative research area in psychology that was once affiliated with psychometrics but that has from the 1960s onward developed independently (Van Zandt & Townsend, 2012). Both mathematical psychology and psychometrics heavily rely on mathematics and are based on models, but the use and interpretation of the models differ strongly. Models in mathematical psychology try to uncover laws or mechanisms that describe human behavior, mostly in the areas of perceptive and cognitive processes (Batchelder, 2010). So rather than a focus on individual differences and measurement, mathematical psychology aims at developing formal psychological theory about cognitive processes. The concept of truth is therefore more a priority for mathematical psychology than it is for psychometrics. An important nuance here is that mathematical psychology possibly also adheres to Gigerenzer’s (1991) tools-to-theory heuristics, which specify that many cognitive theories are inspired by the tools that scientists use in the process of doing research, such as statistics or computers. From that perspective, it could be said that theories developed in mathematical psychology are not only driven by truth but also depend on practical, or utilitarian, aspects and are in fact also a case of reverse engineering. However, whereas mathematical psychologists aim for formal theories that describe specific cognitive processes, psychometric models often have a more data-analytic purpose and are easily transferable to all sorts of substantive or practical matters in (and even outside of) psychological research. The notion of truth is therefore not as leading in psychometrics as it is in mathematical psychology, whereas the instrumentalist view—the goodness of a model is not in its ability to reflect reality but rather in its usefulness for various applications—corresponds more closely with modeling in psychometrics.

Psychometrics has been used throughout its history to solve practical or social problems. Although the nature of these problems has certainly changed (e.g., psychometric instruments are no longer used to select people with higher eugenic value), psychometrics is still used for a broad range of selection and diagnostic purposes. The difference in the moral code between early and contemporary psychometrics shows that the instrumentalist rhetoric of psychometrics is in fact a moral value: The kind of questions that are answered in psychometric research and the specific purpose that psychometric instruments are used for is a matter of what is considered valuable in a specific day and age and thus subject to change. An interesting example in contemporary psychometrics that shows how the purpose of psychometric models are in flux is the rise of diagnostic classification models, a type of latent-class model that can be used to diagnose the type of skills that a student has or has not achieved (Rupp & Templin, 2008). Rather than using estimates of ability as a means for selection, these models are often intended to provide teachers with additional diagnostic information about a student’s skill set—a considerable shift in purpose with regard to the more traditional selection purpose.

## What Is to Be Done?

Early psychometricians were devoted to a new social order: Society would be redesigned on the basis of the measurement of people’s innate abilities so that people would hold the positions they were meant to hold. The political and social motives of psychometrics have gradually faded over time, and in contemporary psychometrics—through its devotion to technological solutions and its absence from public debates—the illusion is created that psychometric research is free from any such values. In this article, we have argued that this idea is incorrect. Several values permeate psychometric research: a commitment to quantity, objectivity, fairness, and utility. Moreover, these commitments are not purely epistemic but have a distinctive moral dimension. Together they position psychometrics as an indispensable tool of a meritocratic society. What are the upshots of this argument?

First, it shows a practical untenability of a certain version of the value-free ideal. For the sake of good research, this idea holds that moral values should not have a direct or indirect effect on any part of developing good knowledge (Reichenbach, 1951). Over the past decades, the value-free ideal has been disputed by several authors (Betz, 2013; Douglas, 2009; Elliott, 2017). Nonepistemic values are not only inescapable but also can even be defensible and desirable when made responsibly (Anderson, 2004). This point has not been made about psychometrics, and we have shown that the four values are intimately linked with the practical goals of this field. So long as these goals persist, the incorporation of values is intrinsic to doing good research.

It does not follow, however, that these values are immutable and that they can safely stay hidden—the second upshot of our argument. Values in psychometrics need to be systematically evaluated on ethical and epistemic grounds. To show value-ladenness and contingency of these values does not amount to a debunking of psychometrics. We strongly resist simplistic inferences to the moral depravity of psychometrics. However, we ring alarm bells about the moral aloofness and a certain blindness of psychometrics to start a conversation about improving this field. We have shown that there is a progressive element to each of the four values. The commitments to quantity, objectivity, fairness, and utility all respond to specific moral threats; namely, they reject the idea that humans are qualitatively different and that any expert can use personal judgment to rank individuals. But these attractive aspects of psychometrics should be weighed against the less attractive ones, namely that psychometrics has contributed to other types of social unfairness or inequality. The emphasis on a strictly meritocratic society—although seemingly committed to fair treatment and equality—has its own dangers that have been pointed out by several authors (Gordon & Terrell, 1981; Lemann, 1999; Sokal, 1987; Stein, 2014). High test scores have become a more than desirable good in many societies; they are the gateway to receiving top-notch education and being successful in society. The technology of testing has not erased inequality, as some early psychometricians had hoped, but has become part of a social order that is not only determined by ability but also by factors such as socioeconomic status, geographic location, and access to education. There is now a “tyranny of merit,” to use the words of Michael Sandel, and this regime is directly linked to deepening inequality, destruction of communities, and polarization along economic and political lines all over the world (Sandel, 2020). Could there be a psychometrics that resists these trends or at least does not exacerbate them? What sort of psychometrics could support a different vision of success—one in which many forms of life are enabled and celebrated, not just getting to a top school or reaching the top tier in a ranking, however fair and objective the test?

These are hard questions, and we do not pretend to offer answers here. But our discussion does have clear implications for action. If psychometricians follow us in abandoning the value-free image of their work, then the natural next step is to ask whether each of the four values we identified are worth preserving. Should the assumption of a quantitative nature of variation be sometimes modified? Is objectivity as the removal of personal judgment worth amending? Might there be a better conception of fairness? Should models be evaluated on their substantive virtues rather than utility alone, and what even counts as utility worth pursuing? Answers to these questions do not have to be universal, which is why we resist any sweeping recommendations. It is possible to suspend the quantitative assumption for some attributes but preserve it for others. It is also possible to expand the definitions of fairness, objectivity, and utility without committing to adopting any single one of them always and everywhere. It is possible to ask for whom psychometric models should be useful, the educational testing industry or a wider set of stakeholders who might be harmed by the work these models do? In exploring these questions psychometricians should keep an eye on trade-offs between the values they wish to endorse. Not all values can be implemented in the same model, and sacrifices are inevitable. This is why there is no sense in insisting on one particular set of values psychometrics should exemplify. It all depends on the context of a particular research project.

Such a complex calculation of costs and benefits may come across as asking for too much, especially of researchers whose identity is often defined by *not* being ethicists, but we have already shown that this identity is misleading. Psychometrics is already knee-deep in values, so our proposal here is only to make this fact more explicit and more examined. The professional responsibility of the psychometrician is more significant than currently recognized and must encompass reflections on whether the values that animate psychometrics today are the values that should animate psychometrics tomorrow. As we have argued in this article, apparently technical concepts such as DIF and fairness acquire their meaning against a backdrop of usually implicit ideas on how a good test should function or how selection procedures should ideally operate. The background assumptions that guide these judgments of good and bad can be explicated to a much greater degree than is customary in contemporary approaches. This does not mean that psychometrics should seek a one-to-one mapping of techniques to value systems because any one technical concept could undoubtedly be applied in various value systems; rather, it is important to investigate the way in which a technical concept operationalizes core values in such systems to understand the role it plays in society. Psychometric tests and techniques are not used in isolation but typically serve a goal that is defined in larger societal structures that involve social, political, and moral value judgments. Developing connections to such larger structures should be an important topic on the agenda of both psychometrics and society at large.

For example, it would be worthwhile to investigate how encoding different value systems into concepts such as fairness and objectivity would play out in terms of the structure of psychometric models and to what kind of tests the implementation of such structures would lead. In this respect, it is noteworthy that Georg Rasch, one the towering figures in psychometrics, derived his models from philosophical assumptions on the qualities a good measurement system should have (i.e., items should be probabilistically invariant in ordering individuals, and individuals should be probabilistically invariant in ordering items; Rasch, 1960). Most existing psychometric work has followed this lead, but that does not imply that no alternative courses of action are possible. These alternatives should be articulated and evaluated.

Finally, a word on the limitations of the current study: We hypothesized about the value commitments of psychometricians as they appear in the published theoretical work in this field; however, we did not explore the full range of methods that could demonstrate these value commitments or address value judgments in all subfields of psychometrics (only in psychometrics as narrowly defined). We therefore hope that this article can be treated as a stepping stone for further studies on the topic. For example, a qualitative study could shed further light on psychometricians’ attitudes and their perceptions of value judgments in psychometrics, a discourse analysis could highlight how values are embedded in the psychometrician’s language, and a sociological analysis could enlighten psychometricians’ participation in public policy and the interconnection between psychometrics and more substantive areas. The bottom line is that values in psychometrics can be uncovered and scrutinized and that doing so with all the methods available is the responsible thing to do.

## Transparency

*Action Editor:* Richard Lucas

*Editor:* Laura A. King

## Notes

1. This precise definition of “psychometrics” as the technical, model-based discipline (not as psychometric applications) is a strategic choice on our part. The role of value judgments in applications of psychometrics is by now familiar—after all, these applications typically have social motivations to improve education or human-resources management. This is why famous critiques, such as Stephen Jay Gould’s *The Mismeasure of Man* (Gould, 1981), tend to focus on specific applications, for example, in making the connection between racism and intelligence research. So to show that applied psychometrics is value-laden hardly counts as newsworthy. On the contrary, showing value judgments at work in pure theory *is* unexpected and therefore newsworthy.

2. Such a methodology has been adopted in many high-profile studies of values in science, including Anderson (2004), Keller and Longino (1996), Lloyd (1993), and Longino (2020). For example, Lloyd (1993) showed that studies of female orgasm assume that orgasm is necessarily linked to reproduction and in this sense implicitly adopt the value of androcentrism.

3. The Rasch model is a psychometric model for analyzing dichotomous data (which are often correct/incorrect item responses) as a function of a person’s ability and item difficulty and which has been claimed to instantiate measurement on equal footing with that in the natural sciences (Bond & Fox, 2015).

4. Although psychometricians mostly engage in quantitative research and consider that it is important to do so for the reliability of psychological science, we do not mean to say here that psychometricians believe there is no place for qualitative research in psychology.

5. An exception is the field of cognitive diagnosis (Junker & Sijtsma, 2001), which is a model that combines quantitative traits with latent-class models to identify whether a student has acquired a specific skill. Cognitive-diagnosis models have become increasingly popular and are well represented in journals such as *Psychometrika*.

6. See James and Burgos (2020) for the history of the idea of polygenesis, the hypothesis that different races have different origins. Also see Zack (2017) for an extensive overview of the history and philosophy of race.

7. Note that there also exist a number of approaches in psychometrics that aim at quantifying or formalizing human judgment, so that human judgment becomes more reliable (and thus less prone to error). Examples of these approaches are generalizability theory (Cronbach et al., 1963) and interrater reliability (e.g., Cohen, 1960).

8. Test equating is a statistical process to adjust test scores so that scores on different test forms are comparable and can be used interchangeably. See Kolen and Brennan (2014) for a comprehensive overview.

9. Several authors have contested the idea that standardized testing contributed to (racial) equality. For example, Au (2016) considered contemporary standardized testing a “racial project.” From this perspective, unfairness with regard to the test or items themselves might have been extensively researched, but standardized testing as a phenomenon still contributes to racial inequality because the actual sources of inequality between groups are not resolved.

10. Eugenics as described in this article and especially with relation to psychometrics was tied to racism and White supremacy. Note that eugenics still has a particular relevance for bioethics, in which such a relationship with racism is denied. There exists a continuing debate on whether selection for certain traits is in fact desirable in processes such as genetic engineering and genetic enhancement (e.g., Anomaly, 2018; Savulescu & Kahane, 2009).
